# Remote Sensing Image Characteristics and Typical Area Analysis of Taiyuan Xishan Ecological Restoration Area

**DOI:** 10.3390/s23042108

**Published:** 2023-02-13

**Authors:** Wang Tao, Zhang Jin

**Affiliations:** School of Mining Engineering, Taiyuan University of Technology, Taiyuan 030024, China

**Keywords:** Taiyuan Xishan, RGB imagery, CIVE computational model, vegetation cover

## Abstract

The Taiyuan Xishan Ecological Restoration Zone is located in the west of Taiyuan City and belongs to the Xishan Coalfield. Due to the resource development activity of coal mining, which is caused by coal gangue accumulation, surface vegetation degradation, bare surfaces, and other phenomena, it is most common in this area. These have an impact on the surface ecology; however, after ecological restoration, the surface ecology has been greatly improved. There are many extraction models of vegetation coverage based on pixel dichotomology combined with multispectral vegetation index, but we believe that the combination of visible light vegetation index to construct models is relatively unexplored. The main problem of how to use the RGB image data in order to quickly and accurately extract vegetation coverage information is still under investigation and needs researchers’ attention. In this paper, through selecting the vegetation coverage as the evaluation index of ecological restoration effect, a new RGB vegetation coverage CIVE calculation model is innovatively proposed to solve the above problem, and on the basis of this model, the vegetation cover change analysis is carried out in the Xishan ecological restoration area of Taiyuan. According to the analysis of vegetation coverage change, relevant paper data, and the characteristics of multiple historical remote sensing images, the ecological restoration area of Taiyuan Xishan is divided into six typical areas. Through empirical evaluation, we summarize and analyze these six typical areas, which can provide typical demonstration roles for other ecological restoration areas. Our findings suggest that the proposed CIVE model realizes the extraction of vegetation cover information and long-term series dynamic monitoring of vegetation coverage.

## 1. Introduction

With the development of the social economy, the ecological and environmental problems caused by excessive mining of coal have become increasingly prominent, so ecological restoration of coal mining areas has become more and more important. The key to ecological restoration in mining areas is vegetation restoration, and vegetation restoration is the basic work of ecological restoration in mining areas [1]. The fractional vegetation cover (FVC) refers to the percentage of the vertical projection area of the aerial part of vegetation (including leaves, stems, and branches) on the ground per unit area compared with the total area of the statistical area [2]. The vegetation cover has an important indicator effect on regional ecosystem environment change [3], which is an important indicator for evaluating the effect of ecological restoration and can reflect the ecological restoration effect of vegetation in mining areas. The vegetation cover measurement methods mainly include the land surface measurement method and the remote sensing monitoring method [4,5].

The surface survey method mainly includes the visual estimation method, sampling method, instrument method, etc., but its artificial subjectivity is strong, labor intensive, limited by manpower, material resources, and time, and it is difficult to apply in a large area [6,7]. Remote sensing technology provides the possibility to monitor vegetation cover over a large area. Tong et al. [8] used the cell dichotomous model and intensity analysis method to analyze the changes of vegetation cover in Inner Mongolia from 1982 to 2010 at three levels: time interval, vegetation cover grade change, and transformation. Based on the TM remote sensing data, Li et al. [9] improved the model parameter calculation method based on the principle of cell dichotomy and constructed a quantitative extraction model of multispectral vegetation index (NDVI) vegetation coverage, which, in fact, realized the extraction of vegetation coverage in the upper reaches of Miyun Reservoir. The cell dichotomy has the advantages of convenient calculation and high accuracy and is widely used in the existing literature [7].

Hui et al. [10] used Sentinel-2 data to calculate the NDVI of the study area by inversion of remote sensing bands and adopted an empirical comparison approach to study the NDVI distribution characteristics of the Shengli and Pingshuo mining areas. Zhang et al. [11] used ground data and model simulations to quantitatively assess the extent of vegetation recovery and its effect on soil wind erosion in the Three Northern Protection Forest System Construction Project Area from 2000 to 2019 on the basis of vegetation cover and vegetation net primary productivity to characterize the vegetation recovery status and to investigate the vegetation recovery potential. Tian et al. [12] used five periods of Landsat remote sensing images in the Lijiang dam area in 1987, 1991, 1994, 2010, and 2018 as data sources and used the regression trend and concentric circle spatial gradient model to obtain the spatial and temporal variation characteristics of vegetation cover.

Peng et al. [13] used an image-based dichotomous model to estimate the vegetation cover in Hunan Province and analyze its spatial and temporal variation and influencing factors. He et al. [14] constructed the simplified visible light vegetation index (SVVI), combined with the sample statistics method to determine the threshold value, and compared eight common visible light vegetation indices with the supervised classification results of the support vector machine (SVM). The accuracy and applicability of the indices were examined using a confusion matrix, using two typical watersheds on the Loess Plateau as examples. Furthermore, Li et al. [15] used three visible bands of red, green, and blue to calculate the visible-band difference vegetation index (VDVI) for areas covered by salt marsh vegetation. The inverse model was established.

Huang et al. [16] used Landsat remote sensing images from 2000, 2010, and 2020 as data sources, extracted vegetation cover in the upper reaches of the Minjiang River in three periods using an image dichotomous model, combined with topographic factors such as elevation, slope, and slope direction, and analyzed the changes in vegetation cover and topographic differentiation characteristics of the study area from 2000 to 2020. The analysis was carried out to provide data support for ecological conservation and land use planning. Mou et al. [17] took Wenshan Zhuang and Miao Autonomous Prefecture as an example and used MODIS data from 2001 to 2020 to analyze the spatial and temporal changes of vegetation in Wenshan Prefecture using a dichotomous model and a linear regression analysis method, on the basis of which the coefficient of variation was calculated to observe the fluctuation of vegetation changes over the 20-year period in the Prefecture.

Li et al. [18] used the Sen+Mann–Kendall time series trend change detection method based on the annual vegetation cover dataset of the potential occurrence range of desertification in China (PEDC) from 2000 to 2020, constructed by an improved directional model such as an elementary dichotomous model. The spatial and temporal characteristics of the vegetation growth in the PEDC, especially in the forest and grass cover areas, were analyzed using the Sen+Mann–Kendall time series trend change detection method. Similarly, Wang et al. [19] studied the spatial and temporal evolution characteristics of the fractional vegetation cover (FVC) on the Loess Plateau from 2001 to 2019 and analyzed the geographical factors affecting FVC by combining geographic probes and correlation analysis.

There are many extraction models of vegetation coverage based on pixel dichotomology combined with multispectral vegetation index, but few papers have been studied on the combination of visible light vegetation index to construct models. How to use the RGB image data to obtain vegetation cover information is an important issue that is still under discussion and research.

Based on the above problems, this paper uses RGB remote sensing image data to construct seven new vegetation coverage extraction models based on the cell dichotomy method by combining the excess green EXG [20], the green–red vegetation index GRVI [21], the excess green–excess red EXGR [22], the normalized green–blue difference index NGBDI [23], the color index of vegetation extraction CIVE [24], the red–green–blue vegetation index RGBVI [25], and the visible–band difference vegetation index VDVI [26]. The optimal model is selected through accuracy evaluation for subsequent analysis. In this paper, we propose a new CIVE model for calculating RGB vegetation coverage. The proposed CIVE model enables the extraction of vegetation cover information and long-term, series-based dynamic monitoring of vegetation coverage. The major and fundamental contributions of our research are as follows:

- We focus on how to use the RGB image data to obtain vegetation cover information.
- We use the RGB remote sensing image data to construct new vegetation coverage extraction models that are based on the cell dichotomy method by combining different indexes.
- A new RGB vegetation coverage CIVE calculation model is innovatively proposed for using the RGB image data to quickly and accurately extract vegetation coverage information.
- We selected vegetation coverage as the evaluation index of ecological restoration effect, and a new CIVE model for calculating RGB vegetation coverage is proposed.

The rest of the paper’s contents are organized in the following manner: An overview of the study area and data selection is provided in Section 2. Different research methods, along with the one proposed in this paper, are elaborated in Section 3. Mathematical proofs and evaluation metrics are also covered in this section. In Section 4, the analysis of the obtained results is demonstrated. A typical area analysis is described in Section 5 in terms of the obtained outcomes. Finally, concluding remarks and some future research directions are given in Section 6.

## 2. Overview of the Study Area and Data Selection

### 2.1. Overview of the Study Area

The Taiyuan West Mountain is located in the border mountain area at the eastern foot of the central Lüliang Mountain Range, from the Fenhe River in the north to Liuziyu in the south to the Tianlong Mountain, belonging to the middle of the Yellow River Basin in North China. The climate is a northern temperate continental monsoon climate, with little rain and wind in spring, a hot and rainy summer, a short and cool autumn, and a cold and dry winter. The average annual temperature is usually 9 °C, while the average annual frost-free period is approximately 202 days. Moreover, the average annual precipitation is about 460 mm.

Taiyuan West Mountain is the general name of the western suburbs of Taiyuan, which cross the three administrative districts of Taiyuan from north to south. This should be noted, as there were more than 2000 small coal kilns in the past in Wanbailin District alone, which were once a typical representative of “dirty and chaotic” in the eyes of the people of Taiyuan. Nevertheless, it has continuous barren mountains, muddy waterways, and lots of resource waste mines that help to make this one of the largest sources of pollution in Taiyuan City. The Taiyuan Xishan Ecological Restoration Zone extends from Honggou Village in the north, Xiyu Coal Mine Forest Area in the south, Huangpo Village in the east, and Taoxing Village in the west, with a total area of about 10 km².

**Figure 1:**
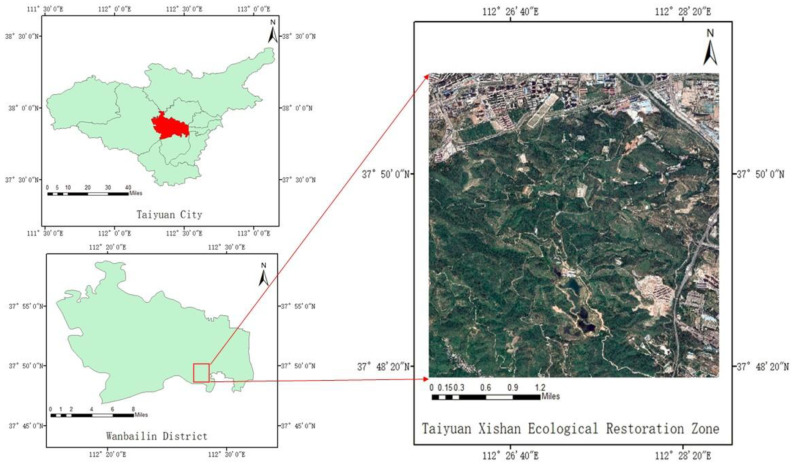
The study area.

Taiyuan Xishan is an important coking coal production base in China. Many years of coal mining have caused sinking, tilting, and deformation of the surface, resulting in goaf areas, collapsed land, gangue mountains, etc. The ecological environment, working environment, and living environment of surrounding residents have been affected. There are two main types of impacts of coal mining on the surface in the Taiyuan Xishan Ecological Restoration Area. The first type is coal mine subsidence (dumps, gangue mountains, etc.) formed by coal mining activities, mainly based on terrain restoration, reclamation, and restoration, and forest land planting. The other type is the closed coal mine, which leaves a large number of industrial relics. The restoration considers cultural, historical, and humanistic factors, adds ideas for landscape design, and transforms the site into new functional areas on the basis of ecological restoration, such as parks, residential areas, museums, tourist attractions, etc. Figure 1 shows the location of the Taiyuan Xishan Ecological Restoration Zone in Wanbailin District, Taiyuan City.

### 2.2. Data Picking

**Figure 2:**
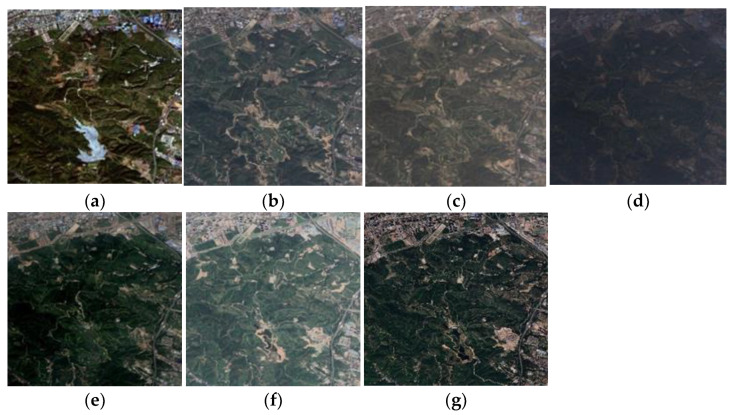
Remote sensing image of the study area. (**a**) 22 September 2010; (**b**) 17 August 2013; (**c**) 16 August 2015; (**d**) 15 September 2016; (**e**) 20 August 2017; (**f**) 20 July 2020; (**g**) 21 June 2021.

Combined with the existing image and governance policies of the Taiyuan Xishan Ecological Restoration Area, the treatment began in 2008. Moreover, the ecological governance goal of “laying the foundation in one year, achieving results in two years, changing the appearance in three years, and becoming a scenic spot in five years” was established, so the image analysis in 2010 and 2013 was selected. At the end of 2014, the goal of full coverage of suitable forest-barren mountains in the Taiyuan Xishan Ecological Restoration Area was completed, so the image analysis in 2015 was selected. From 2016 to the present, the Taiyuan Xishan Ecological Restoration Area has been restored by restoring the damaged surface caused by coal mining, garbage dump treatment, and other measures combined with the existing images, so the images from 2016, 2017, 2020, and 2021 were selected for analysis. We downloaded these seven issues of historical Google Earth high-resolution RGB remote sensing imagery in *.tif format, as shown in Figure 2.

## 3. Research Methods

**Figure 3:**
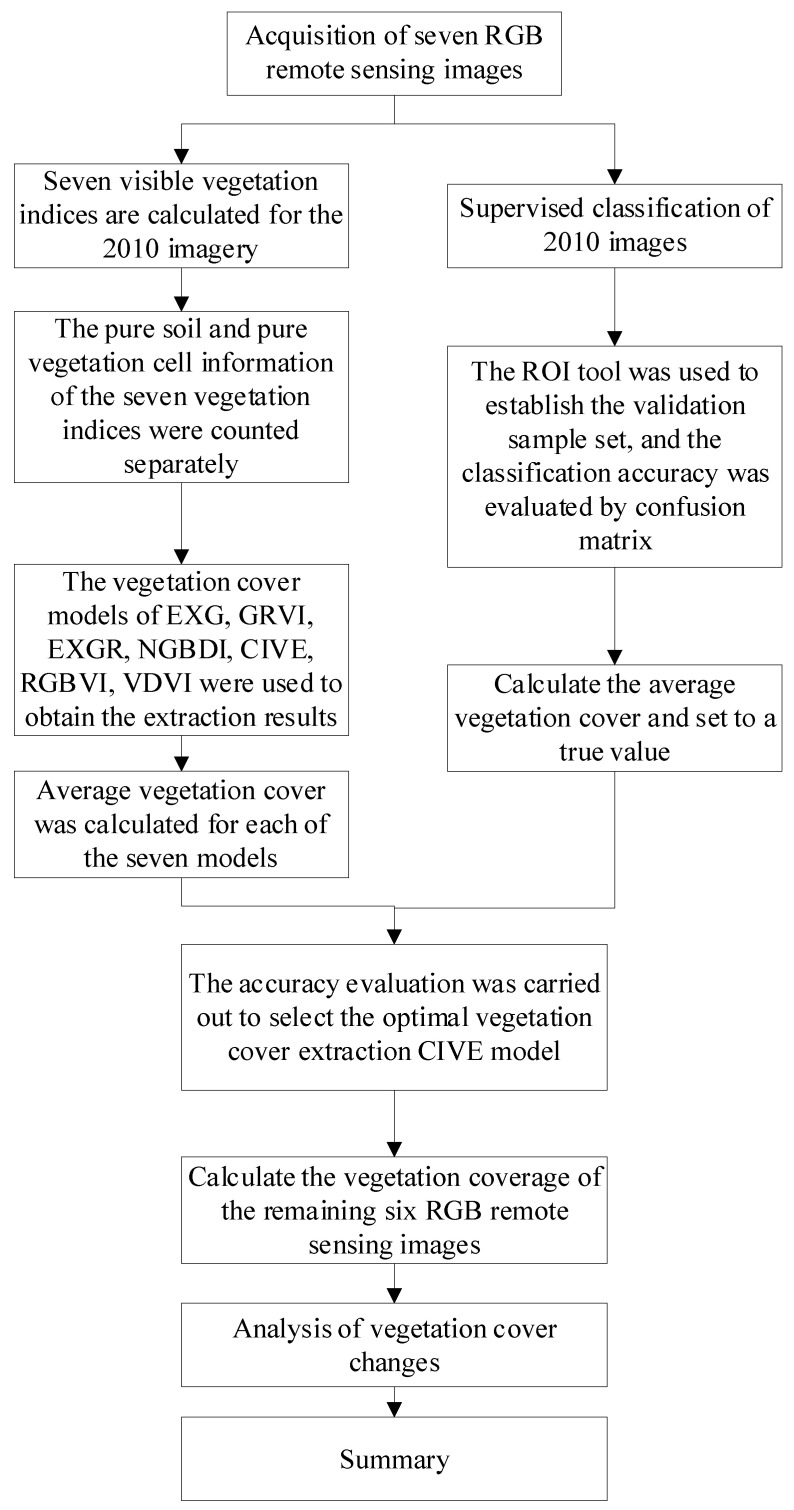
Vegetation coverage change analysis process.

In this study, seven issues of Google Earth’s historical RGB remote sensing images were obtained. We calculated the seven visible light vegetation indices of the 2010 image and counted pure soil and pure vegetation cell information. In the next step, we calculated the excess green (EXG), the green–red vegetation index (GRVI), the excess green–excess red (EXGR), the normalized green–blue difference index (NGBDI), the color index of vegetation extraction (CIVE), the red–green–blue vegetation index (RGBVI), and the visible–band difference vegetation index (VDVI). In the subsequent step, we extracted the seven planting cover extraction models in order to obtain the extraction results and, then, calculated the average vegetation cover. Furthermore, the 2010 images were supervised and classified in order to obtain the average vegetation coverage. Then, accuracy verification was carried out, and the CIVE vegetation cover calculation model with the highest accuracy was selected. Finally, the CIVE vegetation coverage model was used to calculate the remaining six RGB remote sensing images, and the results were analyzed. The overall process and the flowchart of the proposed CIVE model are shown in Figure 3.

### 3.1. Visible Light Vegetation Index

The vegetation index is a simple, effective, and empirical measure of the state of land surface vegetation, which is widely used in global and regional land cover, vegetation classification, and ecological environment change. In this experiment, seven commonly used vegetation indices were introduced (see Table 1), namely the commonly used excess green EXG, the green–red vegetation index GRVI, the excess green–excess red EXGR, the normalized green–blue difference index NGBDI, the color index of vegetation extraction CIVE, the red–green–blue vegetation index RGBVI, and the visible–band difference vegetation index VDVI (see Table 1).

### 3.2. Cell Dichotomy

The cell dichotomy [9,27] is a simple and practical linear model that assumes that the surface information of a cell consists of two parts: (i) pure vegetation; and (ii) pure soil (non-vegetation) information. This relationship is mathematically illustrated as given in Equation (1).

(1) $$F_{FVC}=\frac{V_{I}-V_{I\;soil}}{V_{I\;veg}-V_{I\;soil}}$$

In the above formula, $F_{FVC}$ is a vegetation cover; $V_{I}$ is the vegetation index value of the cell; $V_{I\;soil}$ is the bare soil or no vegetation cover cell vegetation index value; and $V_{I\;veg}$ is the $V_{I}$ value of a 100% pure vegetation cell.

### 3.3. Vegetation Cover Extraction Model

According to the principle of cell dichotomy, the EXG value of a cell can be expressed as the sum of pure vegetation cell information and pure soil cell information. Therefore, substituting Equation (1) can be converted, subsequently, into the EXG vegetation cover extraction model, as given by Equation (2):

(2) $$FVC_{EXG}=\frac{EXG-EXG_{soil}}{EXG_{veg}-EXG_{soil}}$$

where the $FVC_{EXG}\;$is the vegetation coverage calculated by the EXG index; EXG is the value of the EXPONENT of the cell; $EXG_{soil}$ is the value of the cell EXG index on bare soil or without vegetation cover; and $EXG_{veg}$ index value for 100% pure vegetation cells.

Similarly, substituting various indexes such as GRVI, EXGR, NGBDI, CIVE, RGBVI, and VDVI, whose mathematical formulas are given in Table 1, into Equation (1), respectively, can obtain the following six vegetation coverage extraction models (from Equation (3) to Equation (8)). This should be noted because the vegetation coverage is extracted according to the models as given in Formulas (2) to (8), i.e., the vegetation coverage extraction model and pure pixel information.

(3) $$FVC_{GRVI}=\frac{GRVI-GRVI_{soil}}{GRVI_{veg}-GRVI_{soil}}$$

where FVC_GRVI is the vegetation cover calculated from the GRVI index; GRVI is the GRVI index value of the image; GRVI_soil is the GRVI index value of the bare soil or no vegetation cover image; and GRVI_veg is the GRVI index value of the 100% pure vegetation image.

(4) $$FVC_{EXGR}=\frac{EXGR-EXGR_{soil}}{EXGR_{veg}-EXGR_{soil}}$$

In the above equation, FVC_EXGR is the vegetation cover calculated from the EXGR index; EXGR is the EXGR index value of the image; EXGR_soil is the EXGR index value of the bare soil or no vegetation cover image; and EXGR_veg is the EXGR index value of the 100% pure vegetation image.

(5) $$FVC_{\mathrm{NGBDI}}=\frac{NGBDI-NGBDI_{soil}}{NGBDI_{veg}-NGBDI_{soil}}$$

where FVC_NGBDI is the vegetation cover calculated from the NGBDI index; NGBDI is the NGBDI index value of the image; NGBDI_soil is the NGBDI index value of the bare soil or no vegetation cover image; and NGBDI_veg is the NGBDI index value of the 100% pure vegetation image.

(6) $$FVC_{CIVE}=\frac{CIVE-CIVE_{soil}}{CIVE_{veg}-CIVE_{soil}}$$

where FVC_CIVE is the vegetation cover calculated from the CIVE index; CIVE is the CIVE index value of the image; CIVE_soil is the CIVE index value of the bare soil or no vegetation cover image; and CIVE_veg is the CIVE index value of the 100% pure vegetation image.

(7) $$FVC_{RGBVI}=\frac{RGBVI-RGBVI_{soil}}{RGBVI_{veg}-RGBVI_{soil}}$$

In the above formula, the FVC_RGBVI is the vegetation cover calculated from the RGBVI index; RGBVI is the RGBVI index value of the image; RGBVI_soil is the RGBVI index value of the bare soil or no vegetation cover image; and RGBVI_veg is the RGBVI index value of the 100% pure vegetation image.

(8) $$FVC_{VDVI}=\frac{VDVI-{\mathrm{VDVI}}_{soil}}{{\mathrm{VDVI}}_{veg}-{\mathrm{VDVI}}_{soil}}$$

where FVC_VDVI is the vegetation cover calculated from the VDVI index; VDVI is the VDVI index value of the image; VDVI_soil is the VDVI index value of the bare soil or no vegetation cover image; and VDVI_veg is the VDVI index value of the 100% pure vegetation image.

### 3.4. Vegetation Coverage Extraction from RGB Images

We calculated the seven visible light vegetation indices in the image, as described in the literature by Li et al. [9]. In the next step, we took 2% confidence as the pure pixel information of each vegetation index through repeated comparison (that is, the cumulative pixel value of the vegetation index is pure soil cell information, and 98% is pure vegetation cell information). Similarly, the vegetation coverage was extracted according to the models as given by Formulas (2)–(8), i.e., the vegetation coverage extraction model and pure pixel information. Moreover, the supervised classification was carried out in combination with Formula (9) to extract vegetation coverage, and the obtained results were used as truth values to verify the accuracy of the vegetation coverage extraction results of the seven models.

The vegetation cover of the supervised classification results was obtained by the following evaluation metric, as given by Equation (9):

(9) $$FVC_{SC}=\frac{N_{veg}}{N_{veg}+N_{land}+N_{road}+N_{pool}+N_{building}}$$

where $N_{veg}$ is the number of vegetation cells, $N_{land}$ is the number of bare ground cells, $N_{road}$ is the number of pavement cells, $N_{pool}$ is the number of fly ash sedimentation pool cells, and $N_{building}$ is the number of building cells.

### 3.5. Accuracy Evaluation of Vegetation Cover

At present, the photographic method is often used to evaluate the accuracy of vegetation coverage, and because field photography is limited by manpower, material resources, and time, this method has a certain impact on the actual scope of the accuracy evaluation of vegetation coverage. Referring to the literature of Meng et al. [28], this paper performs supervised classification to divide RGB images into five categories, extracts the average coverage, verifies the overall accuracy of the average coverage obtained by the seven vegetation coverage extraction models, and calculates the vegetation coverage extraction error ($E_{C}$) as follows in the following Equation (10):

(10) $$E_{C}=\frac{\left|F_{I}-F_{V}\right|}{F_{I}}\times 100\%$$

In the above Equation (8), the variable $F_{I}$ vegetation coverage is obtained by the supervised classification, while the $F_{V}$ vegetation coverage is obtained by the vegetation coverage model of RGB imagery.

## 4. Analysis of the Results

### 4.1. Monitor Classification Results and Evaluations

**Figure 4:**
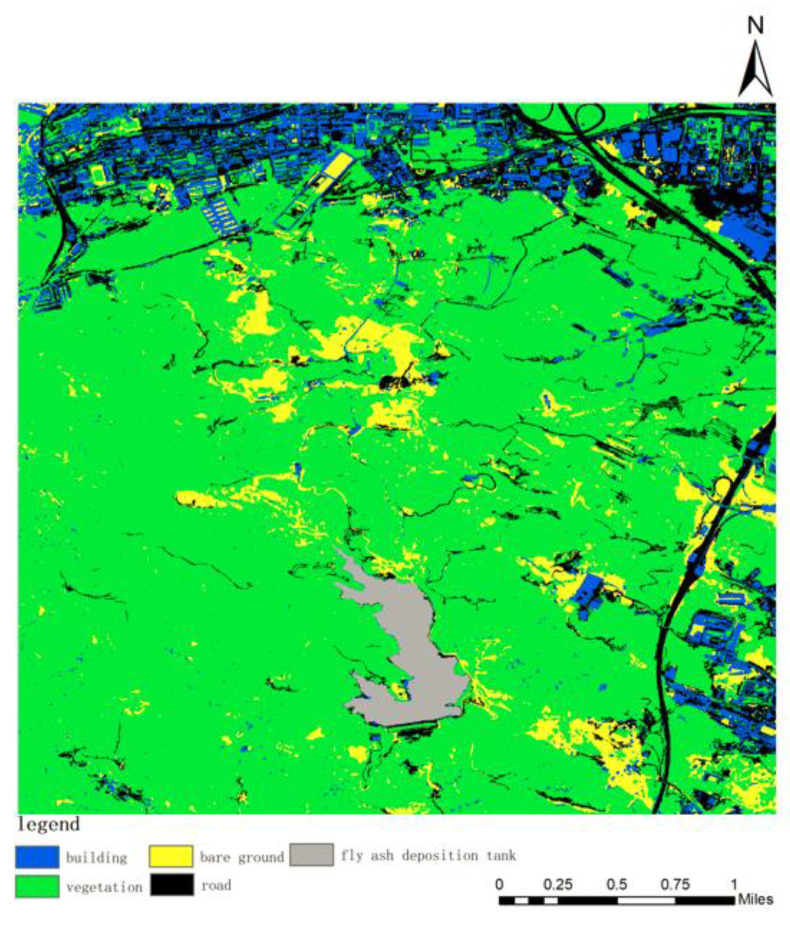
Results of image classification in 2010.

The images of the Taiyuan Xishan Ecological Restoration Area on 22 September 2010 were supervised and classified, and the classification map was obtained, as shown in Figure 4.

The verification sample set is established through the ROI tool, and the sample points marked by themselves are used as the real feature information. Furthermore, approximately 20 sample points are randomly marked for each of the five feature categories, for a total of 100 sample points, and then the classification accuracy is evaluated by using the confusion matrix tool. Subsequently, the total accuracy for the image classification results of the Taiyuan Xishan Ecological Restoration Area on 22 September 2010 is 98.68%, the Kappa coefficient (Kappa) is 98.68%, and the ROI coefficient is 98.18%. After analysis, it can be seen that the classification accuracy is greater than 98% and the classification results have high reliability.

By counting the total number of pixels in each feature category, the results are obtained as shown in Table 2.

Through calculating the proportion of vegetation pixels to total pixels, our evaluation and observation show that the average vegetation coverage is 0.737320, indicating that the overall vegetation coverage in this period is about 73.73%.

### 4.2. Extraction Results and Analysis of Vegetation Cover for Different Models

By counting the pixels of the visible vegetation index image, approximately 2% of the cumulative pixels were selected as pure soil cell information and 98% as pure vegetation cell information. When calculating the vegetation cover, the noise pixel value below the pure soil cell information is classified as 0. Similarly, the noise pixel values, which are higher than the pure vegetation cell information, are classified as 1. The pure soil cell information and the pure vegetation cell information of the seven indices are statistically obtained as shown in Table 3.

In particular, for the vegetation color index (CIVE), the two pieces of information are $CIVE_{\;soil}$ = 0.105882 and $CIVE_{\;veg}$ = −0.819608; in the case of CIVE > 0.105882, FVC takes 0 and is not covered by vegetation; CIVE < −0.819608, 100% is all vegetation cover.

**Figure 5:**
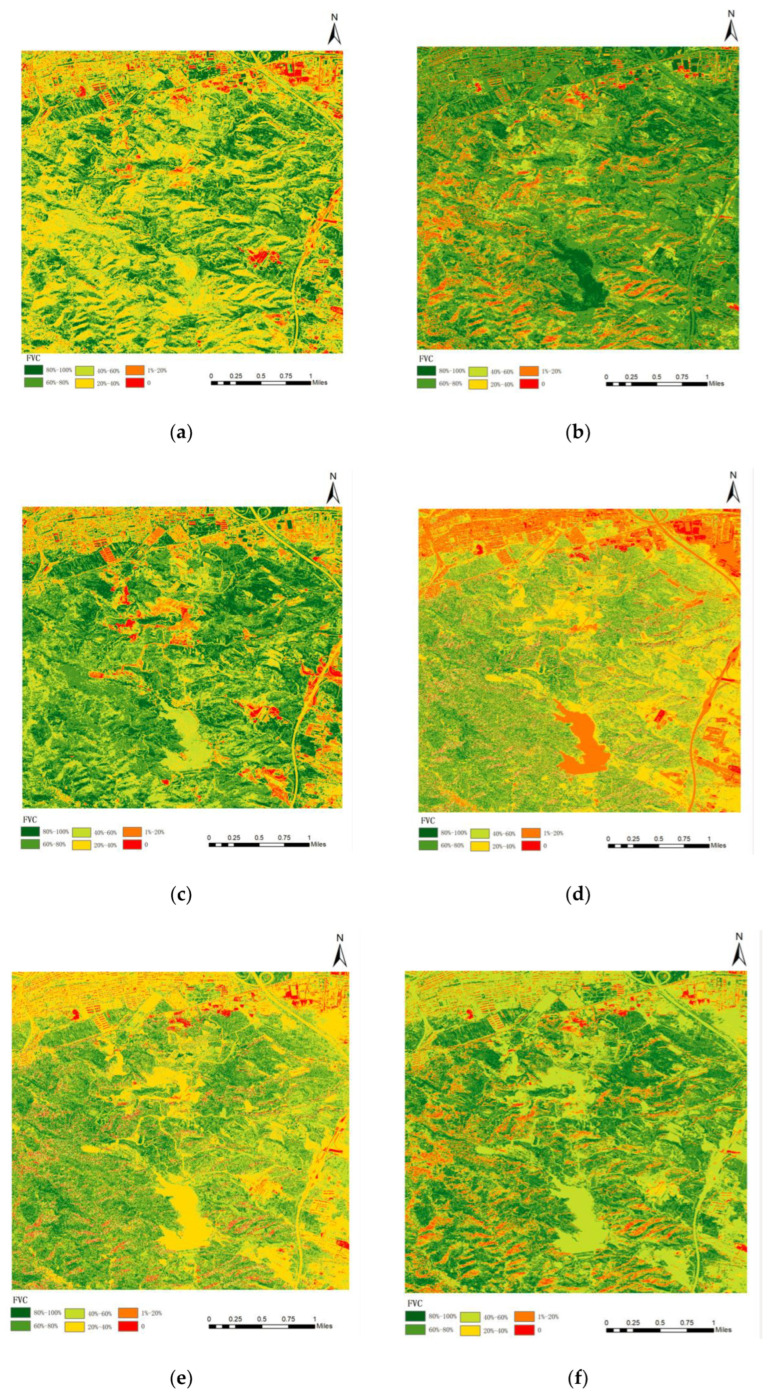

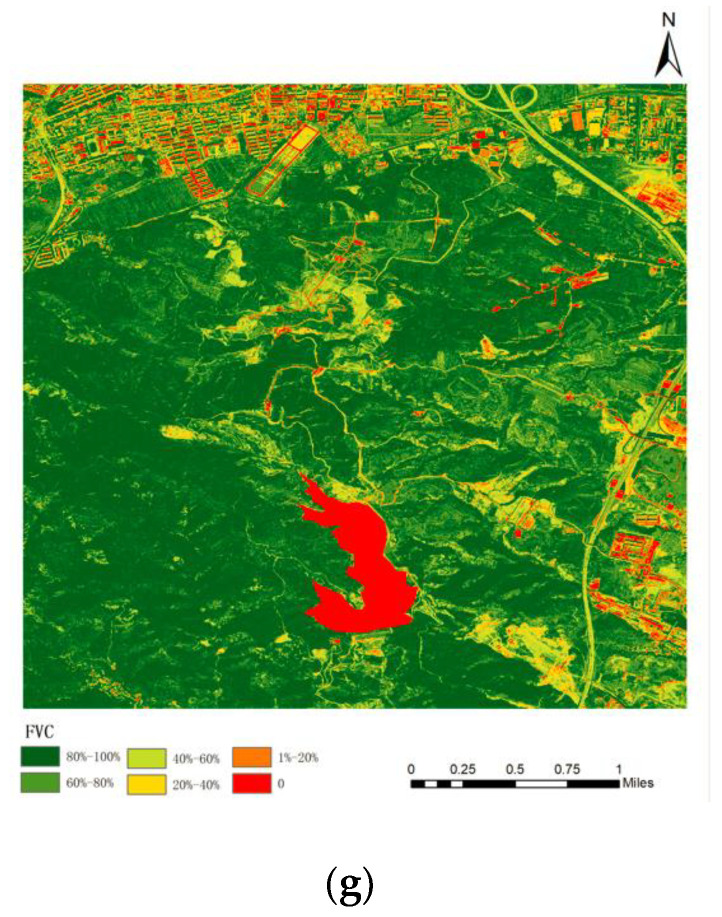
Extraction results of vegetation coverage for different models. (**a**) EXG; (**b**) GRVI; (**c**) EXGR; (**d**) NGBDI; (**e**) RGBVI; (**f**) VDVI; (**g**) CIVE.

According to the vegetation cover extraction model and then according to the five-level classification standards of 0–20%, 20–40%, 40–60%, 60–80%, and 80–100%, the results of the vegetation cover map of the seven models on 22 September 2010 were obtained, as shown in Figure 5.

The average vegetation coverage was statistically obtained, and the accuracy evaluation of the vegetation coverage (Table 4) was carried out. From the outcomes, it can be seen, as shown in Table 4, that among the seven models, the vegetation coverage accuracy obtained by the CIVE vegetation coverage extraction model was the highest.

### 4.3. Vegetation Cover Extraction Results of CIVE Model

**Figure 6:**
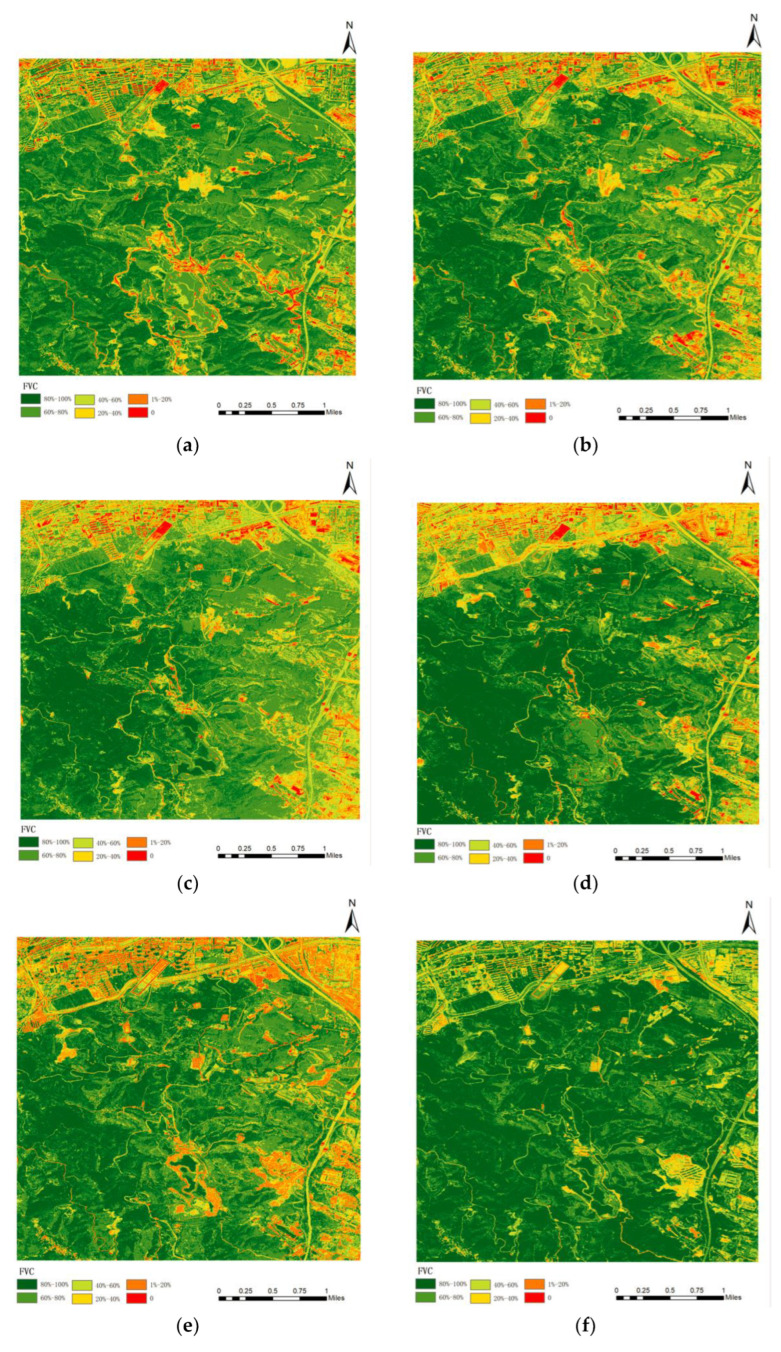
The CIVE model vegetation coverage extraction results. (**a**) 17 August 2013; (**b**) 16 August 2015; (**c**) 15 September 2016; (**d**) 20 August 2017; (**e**) 20 July 2020; (**f**) 21 June 2021.

According to the CIVE vegetation coverage extraction model, the results of the multi-stage vegetation coverage map were obtained according to the six-level classification criteria of 0, 1–20%, 20–40%, 40–60%, 60–80%, and 80–100%, as shown in Figure 6.

### 4.4. Analysis of Vegetation Cover Changes

**Figure 7:**
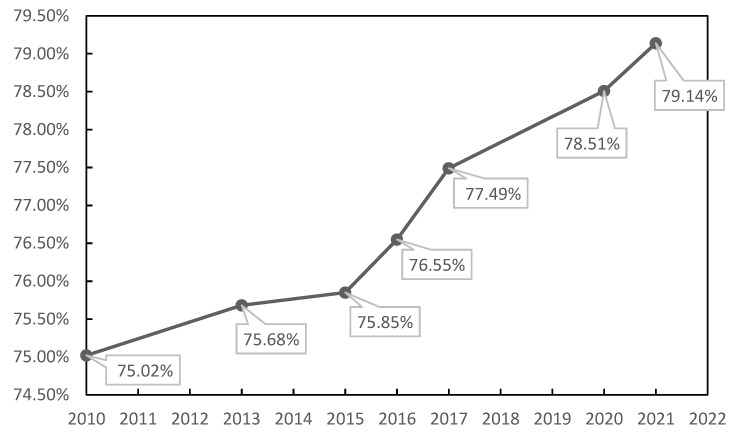
Line chart of vegetation coverage change in CIVE model.

According to the CIVE vegetation coverage model, the average vegetation coverage results obtained by the seven RGB image statistics are calculated, and a line chart of vegetation cover changes is plotted, as shown in Figure 7:

In the previous experiment, the vegetation coverage map of the seven RGB images was calculated according to the CIVE vegetation coverage model. In fact, this reflected the vegetation changes in the study area, and the vegetation coverage of most areas in the study area showed a year-by-year growth trend, from 75.02% in 2010 to 79.14% in 2021. It shows that the government’s implementation of ecological restoration measures in the area has obvious results. The results of Figure 7 show that since 2010, the vegetation coverage began to slowly recover with the implementation of governance policies, increased the fastest in 2015–2017, and slowed down from 2017 to 2021 with the construction of building land in Taiyuan Xishan Ecological Restoration Zone.

The spatial distribution of average vegetation coverage in 2010, 2013, 2015, 2016, 2017, 2020, and 2021 (Figure 6) was comprehensively compared, and the average vegetation coverage value (shown in Figure 7) showed a gradual increase trend. From the spatial distribution map of vegetation coverage, the area variation of vegetation coverage at different grades was different; that is, the area without vegetation cover and the area with low vegetation cover showed a downward trend, while the area with high vegetation cover showed an increasing trend. In general, the overall vegetation coverage of Taiyuan Xishan Ecological Restoration Area is high, and the continuous growth of high vegetation coverage areas indicates that the vegetation quality of Taiyuan Xishan Ecological Restoration Area is constantly improving, and the vegetation restoration trend is significant, which shows that the vegetation coverage in this area has improved significantly through government intervention and ecological restoration intervention.

Although the overall trend of improvement in Taiyuan Xishan Ecological Restoration Area is increasing, it can be found that the vegetation coverage in the southeast corner of the study area has deteriorated due to local changes. This is due to the destruction of the surrounding surface vegetation with the construction of a large number of buildings in the area, which indicates that when building residential and commercial land in the Taiyuan Xishan Ecological Restoration Area, appropriate attention should be paid to protecting the surrounding vegetation to avoid vegetation degradation, and at the same time, it is necessary to increase the greening of vegetation around the building land. This is also an important reference for the further restoration of the Taiyuan Xishan Ecological Restoration Area.

## 5. Typical Area Analysis

The Taiyuan Xishan Ecological Restoration Area is mainly affected by coal mining, and due to its wide range, it is related to its original topography and landform, forming a specific landscape form. Later, the area damaged by coal mining was restored by returning farmland to forest and grassland. On this basis, it has been transformed into a leisure and sightseeing tourism place with green space as the mainstay. Moreover, with the goal of creating beautiful mountain gardens and the collection of road construction, afforestation, landscape setting, leisure tourism, and supporting services, the environmental chaos and pollution that caused stubborn diseases in the Taiyuan Xishan Ecological Restoration Area have been effectively controlled [29].

According to the analysis of vegetation coverage change, related paper data [30,31,32,33,34], and the characteristics of multiple historical remote sensing images, the Xishan ecological restoration area of Taiyuan was divided into six typical areas, including: (i) the coal gangue pile treatment area; (ii) the vegetation degradation restoration area; (iii) the ash pond transformation area; (iv) the amusement garden landscape regeneration area; (v) the bare land re-greening area and industrial land reconstruction area; and (vi) these six typical areas were summarized and analyzed. The obtained results and findings are discussed in terms of four major evaluation indicators: (i) Pre-treatment image, geometric features; (ii) Color characteristics; (iii) Texture characteristics; and (iv) Context characteristics.

### 5.1. Gangue Pile Treatment Area

Most of the gangue disposal sites in the Taiyuan Xishan Ecological Restoration Area are dumped from high slopes in the valley and are naturally piled up disorderly in the process of formation, occupying a large amount of land. It causes pollution in the environment and affects the lives of residents. The reconstruction of the coal gangue area in the Taiyuan Xishan Ecological Restoration Area is mainly aimed at its characteristics and forestry-based land reclamation. After the remediation of the treated area platform and slope surface with vegetation conditions in accordance with the principle of “suitable land, suitable for trees,” it has been combined with the tree species and biological characteristics. This is, in fact, done with the help of natural, dense planting techniques that form a stable ecosystem and are combined with artistic expression techniques in order to provide leisure activities for the surrounding residents [29].

Pre-treatment image, geometric features: A large area of irregular flake gangue piles is distributed in this treatment area, among which the road transporting the discharged coal gangue is distributed on the bare land in a strip. Color characteristics: There are black roads in the dark black cinder gangue accumulation area and black-gray bare land in this treatment area, surrounded by dark green vegetation around the gangue. Texture characteristics: The distribution of gangue piles in this treatment area is messy and has no obvious rules, and the texture is rough. Context characteristics: The vegetation was dominated by woodland before and after the governance area.

**Figure 8:**
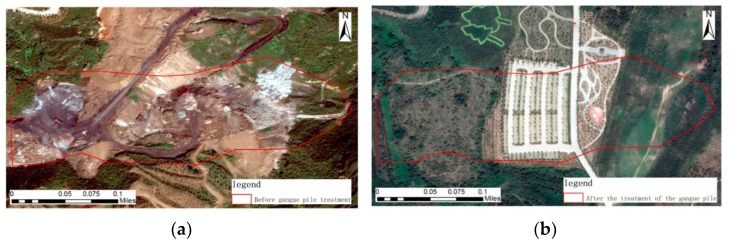
Remote sensing images before and after ecological restoration in the coal gangue treatment area. (**a**) before treatment; (**b**) after treatment.

Imagery after treatment, geometric features: Parking lots with regular geometry covered by sheet vegetation in the area, and strips of roads of different widths around the parking lots. Color characteristics: The parking lots, landscape viewing paths, and wide cement avenues at the entrances and exits of the parking lots in this area are gray-white, and the surrounding vegetation cover is different but overall light green. Texture characteristics: There are plantations arranged in a consistent direction and approximately parallel arrangement in this area, and the vegetation distribution in other parts is sparse, without obvious rules, and the texture is rough. Context characteristics: The upper part of the gangue pile treatment area has roads, sparse vegetation, and high cover vegetation, and the lower part is dominated by dark green woodland, as shown in Figure 8.

### 5.2. Vegetation Degradation Restoration Area

Pre-governance imagery, geometric features: The sparse vegetation in the pre-governance image has multiple strips of roads and a piece of irregularly shaped bare land on which there are multiple rectangular buildings with regular geometric features. Color characteristics: The large areas of black-gray bare land with sparse dark green sparse vegetation, light blue, and white buildings above the light gray road. Texture characteristics: The sparse vegetation in this area is arranged in rows and rows, with obvious texture characteristics, while the rest of the vegetation is messy and has no obvious features. Context characteristics: The area is predominantly woodland vegetation.

**Figure 9:**
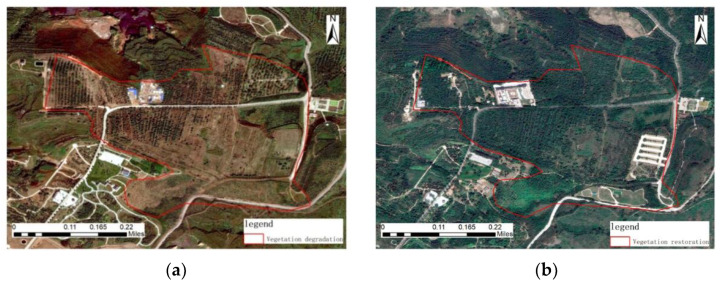
Remote sensing images before and after ecological restoration in the vegetation degradation restoration area. (**a**) before treatment; (**b**) after treatment.

Post-governance image, geometric features: Multiple regularly arranged rectangular buildings on the original bare land are remediated as a campus, and a parking lot with regular geometry is built in the southeast of the area on top of the original sparse vegetation. Color characteristics: Large areas of black and gray bare land are treated with lush dark green vegetation and good vegetation. Texture characteristics: We observed that the distribution of features in this area is not obvious, and the texture is rough. Context characteristics: The area is predominantly densely forested vegetation, as shown in Figure 9.

### 5.3. Sediment Tank Renovation Area

For a long time, Shichagou, where the Taiyuan Xishan Ecological Restoration Zone is located, has been used as a fly ash sedimentary pond. The sedimentation pond covers an area of about 700 acres, and the total amount of fly ash dumped reached 11.2 million tons, causing serious pollution and damage to the local groundwater and surface vegetation. After geological investigation, expert demonstration, and safety assessment, the method of directly driving anti-slip piles into the rock layer for all dam bodies is first adopted to ensure the safety of the dam body. Since then, they have cleaned up 2 million cubic meters of fly ash, backfilled more than 1.5 million cubic meters of earth, treated the bottom of the lake using the most advanced geotextile leakage prevention technology, and finally turned the fly ash sedimentary pond into a green ecological lake.

Pre-governance image, geometric features: The area is a sedimentary pool with irregular geometry and no obvious geometric features. There is a strip of dirt road around the sedimentary pool. Color characteristics: There is a large area of light gray sedimentary pooling in the image before treatment, surrounded by dark yellow bare land, accumulations of dark black gangue, and dark yellow roads with clear boundaries. Texture characteristics: The distribution of features in this area is not obvious, and the texture is rough. Context characteristics: The area is dominated by dark green woodland.

**Figure 10:**
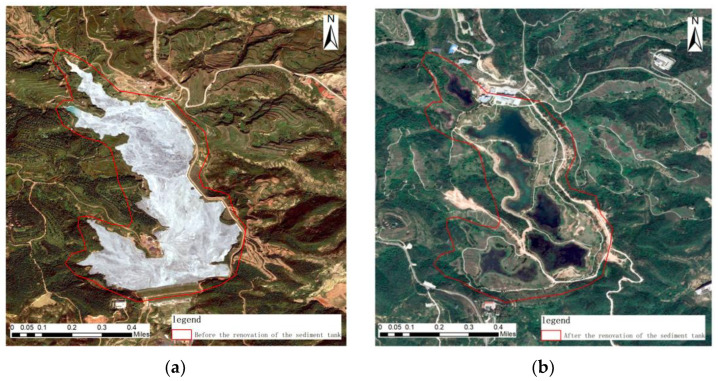
Remote sensing images before and after ecological restoration in the ash pool reconstruction area. (**a**) before treatment; (**b**) after treatment.

Post-treatment image, geometric features: After the treatment, irregular-block artificial ecological lakes were distributed in this area, and building land with regular geometric shapes was built in the upper part of the artificial lake. Color characteristics: The area is dominated by dark green artificial ecological lakes, surrounded by a low degree of light green vegetation, and some light gray roads are distributed with light yellow bare land. Texture characteristics: The distribution of features in this area is not obvious, and the texture is rough. Context characteristics: The area is predominantly densely forested vegetation, as shown in Figure 10.

### 5.4. Amusement Park Landscape Regeneration Area

The landscape design of the park is mainly based on the topographic conditions of the low-lying land, which were caused by the original coal mining. Therefore, slope treatment is carried out to form a water feature, and “Muxi Gallery“ pavilions and small bridges are built on the water’s surface. The water surface is divided by plants and treated with soft slopes. On the south side of the “Muxi Gallery,“ a large area of lawns and small walking paths form a beautiful scenery, and in the landscape configuration, “joe, shrub, grass” combined with the stone landscape of classical gardens are combined to set up, and the surrounding pavilions, corridors, and bridges are combined to form a fresh and elegant environment. The amusement park occupies the largest area in the Wan Berlin Ecological Park, in which the spiritual “water” is used as the main center point of the landscape. Standing in the gallery, you can enjoy the fragrant beauty of the park [29].

Pre-governance imagery, geometric features: The pre-governance imagery has irregular blocks of sparse vegetation areas and bare land distribution in the middle of multiple strip roads. Color characteristics: There is green woodland in the light gray roads distributed in the network, dark yellow bare land around the woodland, and the degree of vegetation cover is average. Texture characteristics: There are no obvious regularities in the figures of the image, and the texture is rough. Context characteristics: The upper part of the area was covered by sparse the light-green vegetation before treatment, and the dark-yellow bare land was mainly below.

**Figure 11:**
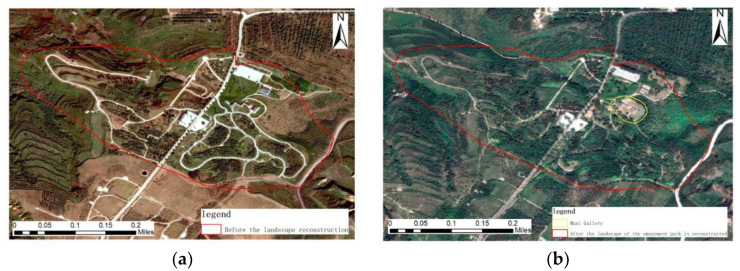
Remote sensing images before and after ecological restoration in the garden landscape reconstruction area. (**a**) before treatment; (**b**) after treatment.

Post-governance imagery, geometric features: The block boundaries between vegetation areas are not obvious, striped roads are obscured by dense vegetation, and geometric features are not obvious. Color characteristics: The area is dominated by dark green woodland, where there are several rectangular gray-white buildings, and the roads are gray and distributed among the vegetation. Texture characteristics: There are no obvious regularities in the figures of the image, and the texture is rough. Context feature: The area in the image is predominantly densely wooded, as shown in Figure 11.

### 5.5. Bare Land Regreening Area

Pre-governance image, geometric features: In the pre-governance image, irregular blocks of bare land are the main ones, and strips of roads are distributed on the bare land. Color characteristics: The gray-white network roads are distributed on dark-red bare land without green vegetation cover. Texture characteristics: There are no obvious regularities in the figures of the image, and the texture is rough. Context characteristics: The area in the image is predominantly sparse vegetation with low coverage.

**Figure 12:**
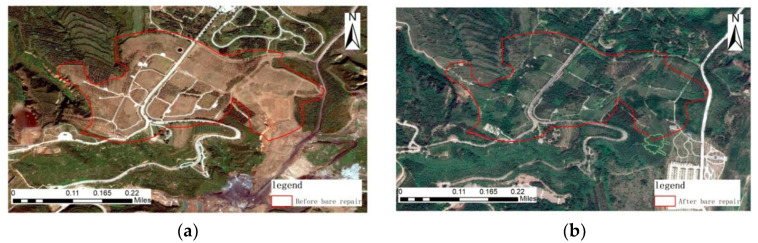
Remote sensing images before and after ecological restoration of the bare land regreening area. (**a**) before treatment; (**b**) after treatment.

Post-governance image, geometric features: The post-governance image is dominated by irregular block vegetation areas, and strip-like roads are distributed in the block vegetation. Color characteristics: The light gray network roads distribute vegetation with different coverage, with light green blocks of sparse forest and dark green, leafy planted land. Texture characteristics: The artificial vegetation texture in this area has obvious regularity, is arranged neatly along a certain texture direction, and is distributed in blocks. Context characteristics: The area in the image is predominantly covered by vegetation, as shown in Figure 12.

### 5.6. Industrial Land Redevelopment Area

Pre-treatment image, geometric features: There are several rectangular shapes of plant buildings in the pre-treatment image, irregular lumpy gangue piles around the plant building, and multiple strip roads between the plant buildings and around the plant buildings. Color characteristics: The dark black gangue piles and black-gray bare land are distributed in a large area of light blue factory buildings, mixed with sparse green vegetation. Texture characteristics: There are no obvious regularities in the figures of the image, and the texture is rough. Context characteristics: The area in the image is dominated by light green vegetation with low coverage.

**Figure 13:**
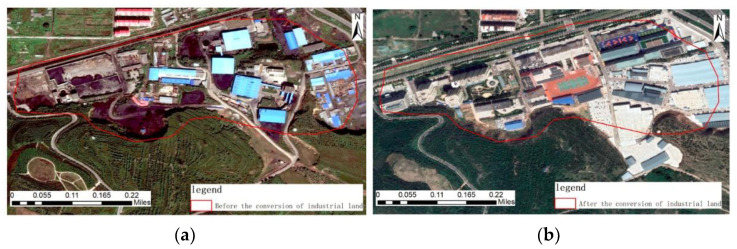
Remote sensing images before and after ecological restoration in an industrial land reconstruction area. (**a**) before treatment; (**b**) after treatment.

Post-treatment image, geometric features: The large area of factory buildings and gangue accumulation areas that existed in the post-treatment image disappeared and was replaced by building land, including residential areas, schools, transportation bureaus, etc. Color features: The color features are not obvious in the image after governance. Texture characteristics: There are no obvious regularities in the figures of the image, and the texture is rough. Context feature: The area in the image is predominantly green vegetation, as shown in Figure 13.

## 6. Conclusions and Future Work

In this paper, vegetation coverage is selected as the evaluation index of ecological restoration effect, and a new CIVE model for calculating RGB vegetation coverage is proposed. The proposed CIVE model enables the extraction of vegetation cover information and long-term, series-based dynamic monitoring of vegetation coverage in this area. According to the relevant paper data and remote sensing image characteristics, the Taiyuan Xishan Ecological Restoration Area was divided into six typical areas, including: (i) the coal gangue pile treatment area; (ii) the vegetation degradation restoration area; (iii) the sediment pond transformation area; (iv) the amusement garden landscape regeneration area; (v) the bare land regreening area; and (vi) the industrial land reconstruction area. Moreover, these six typical areas were summarized and empirically analyzed. The Vegetation Condition Index (VCI) can also be added as a value addition to the work because it compares the current NDVI with the range of values observed for the same period in previous years. This will give a very clear concept for implementing the proposed model in the real world, and we will keep it for our future work. The analysis and our evaluation led to the following conclusions and findings:

1. The ecological restoration and governance of mining areas should adhere to the principle of taking into account the development of mineral resources and ecological environmental protection. Moreover, efforts should be made to strengthen the ecological restoration measures in the process of coal mining, adopt measures of “mining and treatment,” and practice the development idea that “green water and green mountains are gold and silver mountains”.
2. By studying the change in vegetation coverage in the Taiyuan Xishan Ecological Restoration Area, the quality of the vegetation in the Taiyuan Xishan Ecological Restoration Area is constantly improving, and the vegetation restoration trend is also significant. In essence, in fact, this shows that through government intervention and ecological restoration intervention, the vegetation coverage in this area has improved significantly.
3. Through the long-term dynamic monitoring of the vegetation coverage in the Xishan ecological restoration area of Taiyuan, it can be seen that the ecological restoration governance has multiple treatments in the same area. Therefore, the possibility of obtaining ideal results after one treatment is quite low, and it is necessary to continuously explore and investigate the governance model suitable for Xishan.
4. According to the relevant paper data and remote sensing image characteristics, the Taiyuan Xishan Ecological Restoration Area is divided into six typical areas, including: (i) the coal gangue pile treatment area; (ii) the vegetation degradation restoration area; (iii) the sediment pond transformation area; (iv) the amusement garden landscape regeneration area; (v) the bare land regreening area; and (vi) the industrial land reconstruction area. While restoring the ecological environment, it has produced certain economic and social benefits, which can provide a reference for subsequent ecological restoration work.
5. When building on residential and commercial land in the Taiyuan Xishan Ecological Restoration Zone, attention should be paid to protecting the surrounding vegetation to avoid vegetation degradation. Furthermore, at the same time, it is also necessary to increase the greening of vegetation around the building land, which is also an important reference for the further restoration of the Taiyuan Xishan Ecological Restoration Area.
6. Due to the limitations of the data selected for this study, further research is needed to validate the CIVE model based on UAV visible wavelength images and improve it to enhance its adaptability and form a more complete technical process and methodological system.

## Figures and Tables

**Table 1:** Commonly used visible vegetation index.

Vegetation Index	Full Name	Formula	References
EXG	Excess green	2 × G − R − B	[10]
GRVI	Green–red vegetation index	(G − R)/(G + R)	[11]
EXGR	Excess green–excess red	EXG − (1.4 × R − G)	[12]
NGBDI	Normalized green–blue difference index	(G − B)/(G + B)	[13]
CIVE	Color index of vegetation extraction	0.44 × R + 0.88 × G + 0.39 × B + 18.79	[14]
RGBVI	Red–green–blue vegetation index	(G^2^ − (R × B))/(G^2^ + (R × B))	[15]
VDVI	Visible–band difference vegetation index	(G − (R + B)/2)/(G + (R + B)/2)	[16]

where R is the red channel, G is the green channel, and B is the blue channel.

**Table 2:** Figure category pixel count in 2010.

Figure Category	Vegetation	Bare Ground	Road	Fly Ash Deposition Tank	Building
Total number of pixels	13,143,311	1,267,092	1,838,215	320,001	1,257,173

**Table 3:** Figure category pixel count in 2010.

	Cell Information	Pure Soil Cell Information	Pure Vegetation Cell Information
Index			
EXG		−29.552941	50.658824
GRVI		−0.592157	0.223529
EXGR		−101.218803	45.138835
NGBDI		−0.192157	1
RGBVI		−0.458824	1
VDVI		−0.435294	0.427451
CIVE		0.105882	−0.819608

**Table 4:** Accuracy evaluation of different vegetation coverage models.

Vegetation Index Model	Vegetation Cover			Extraction Error/%
	Cell Dichotomy	Supervise Classification	Difference	
EXG	0.514216	0.737320	0.223104	30.26%
GRVI	0.629308	0.737320	0.108012	14.65%
EXGR	0.580621	0.737320	0.156699	21.25%
NGBDI	0.434810	0.737320	0.302510	41.03%
RGBVI	0.509429	0.737320	0.227891	30.91%
VDVI	0.570686	0.737320	0.166634	22.60%
CIVE	0.750179	0.737320	0.012859	1.74%

## Data Availability

Not applicable.
